# Challenges in estimating the prevalence of vitamin D deficiency in Africa

**DOI:** 10.1016/S2214-109X(22)00040-7

**Published:** 2022-04-01

**Authors:** Reagan M Mogire, Sarah H Atkinson

**Affiliations:** Kenya Medical Research Institute (KEMRI), Centre for Geographic Medicine Research—Coast, KEMRI– Wellcome Trust Research Programme, Kilifi 80108, Kenya; Kenya Medical Research Institute (KEMRI), Centre for Geographic Medicine Research—Coast, KEMRI– Wellcome Trust Research Programme, Kilifi 80108, Kenya; Department of Paediatrics, University of Oxford, Oxford, UK

## Authors’ reply

We thank Aiyong Cui and colleagues for their interest in our systematic review of vitamin D deficiency in Africa.^1^ Cui and colleagues identified three studies in which we mistakenly included the recruited number of participants rather than the number with vitamin D measurements (PMID 24605693, 30822819, and 31159206). Correcting these mistakes did not alter prevalence estimates in the revised meta-analyses. Cui and colleagues also identified data extraction errors for three studies, which changed prevalence estimates. In two studies, we mistakenly misclassified participant subgroups (PMID 30866564 and 26070223), and in a third study (PMID 30375272), we made an error in extracting the prevalence of vitamin D deficiency, defined as 25-hydroxyvitamin D (25[OH]D) of less than 50 nmol/L, which resulted in minor changes to overall and subgroup estimates (table). In our initial study design, we considered using 25(OH)D concentrations of 30–50 nmol/L, 50–75 nmol/L, and 75–150 nmol/L, as suggested by Cui colleagues, but few studies from Africa included these cutoffs.

We took the opportunity to repeat our searches and analyses and identified an additional study by Laird and colleagues^2^ from the Seychelles, which was not included in our original publication. We have added estimates from that study, in addition to the corrections suggested by Cui and colleagues, to the final corrected manuscript.

After correcting our meta-analyses by addressing the errors highlighted by Cui and colleagues and adding the study by Laird and colleagues, the overall prevalence of vitamin D deficiency, as defined by 25(OH)D levels of less than 50 nmol/L, was revised from 34·22% to 34·18%; the prevalence as defined by 25(OH)D of less than 30 nmol/L was revised from 18·46% to 17·31%; and the prevalence as defined by 25(OH)D of less than 75 nmol/L was revised from 58·83% to 58·54% (table). Some prevalence estimates by subgroup, and the estimates of mean 25(OH)D concentration overall and in some subgroups, were also revised (table). The conclusions of our study remain unchanged after implementing these corrections.

## Figures and Tables

**Table:** Corrections to estimates of prevalence of vitamin D deficiency and mean 25(OH)D concentration

	Overall	Newborn babies	Children	Adults (non-pregnant)	Pregnant women
**Prevalence of vitamin D deficiency by 25(OH)D cutoff, % (95% CI)**					
<50 nmol/L					
Previous estimate	34·22% (26·22–42·68)	49·07% (24·88–73·49)	22·99% (12·03–36·14)	35·62% (24·56–47·50)	43·91% (15·14–75·07)
Revised estimate	34·18% (26·30–42·51)	49·07% (24·88–73·49)	25·38% (l3·71–39·12)	33·96% (23·l3–45·69)	43·50% (l7·18–71·92)
<30 nmol/L					
Previous estimate	18·46% (10·66–27·78)	63·72% (9·20–100·00)	10·55% (3·25–21·14)	12·59% (4·83–23·16)	52·86% (5·90–96·64)
Revised estimate	17·31% (9·86–26·27)	63·72% (9·20–100·00)	10·55% (3·25–21·14)	12·58% (4·83–23·16)	33·29% (1·66–78·50)
<75 nmol/L					
Previous estimate	58·83% (50·90–66·54)	76·78% (23·65–100·00)	46·42% (31·86–61·28)	59·35% (49·85–68·51)	79·20% (40·28–99·90)
Revised estimate	58·54% (50·23–66·62)	76·78% (23·65–100·00)	44·19% (29·41–59·51)	61·37% (51·54–70·76)	68·62% (28·19–97·12)
**Mean 25(OH)D concentration, nmol/L (95% CI)**					
Previous estimate	67·62 (64·36–70·88)	50·60 (38·91–62·29)	72·22 (64·89–79·54)	69·04 (64·52–73·57)	65·73 (45·65–85·81)
Revised estimate	68·10 (64·83–71·37)	50·60 (38·91–62·29)	72·22 (64·87–79·57)	69·38 (64·82–73·94)	68·46 (49·91–87·01)

25(OH)D=25-hydroxyvitamin D.
